# Feminicídios em Minas Gerais, Brasil: da caracterização dos eventos à análise espaço-temporal

**DOI:** 10.1590/0102-311XPT015224

**Published:** 2025-02-24

**Authors:** Camila Alves Bahia, Isabella Vitral Pinto, Carla Machado da Trindade, Camila Mattarelli de Abreu e Silva, Patricia Habkouk, Taynãna César Simões, Daniel Cardoso Portela Câmara, Paula Dias Bevilacqua

**Affiliations:** 1 Instituto René Rachou, Fundação Oswaldo Cruz, Belo Horizonte, Brasil.; 2 Centro Operacional das Promotorias de Justiça de Combate à Violência Doméstica e Familiar Contra a Mulher, Ministério Público de Minas Gerais, Belo Horizonte, Brasil.

**Keywords:** Violência de Gênero, Análise Espaço-temporal, Política Pública, Segurança, Homicídio, Gender-Based Violence, Spatio-Temporal Analysis, Public Policy, Safety, Homicide, Violencia de Género, Análisis Espacio-Temporal, Política Pública, Seguridad, Homicidio

## Abstract

Os objetivos deste estudo foram caracterizar os feminicídios ocorridos em Minas Gerais, Brasil, e comparar a tendência das taxas de feminicídio e homicídio de mulheres nas 89 microrregiões de saúde do estado. Foi conduzido um estudo transversal do tipo ecológico utilizando dados de feminicídios consumados ocorridos entre 2016-2020, consolidados pelo Ministério Público de Minas Gerais. Foram calculadas as taxas de feminicídio e de homicídio de mulheres por 100 mil pessoas do sexo feminino, segundo as microrregiões de saúde. Para o cálculo da última, utilizou-se os dados públicos do Sistema de Informações sobre Mortalidade. Uma análise foi feita acerca de microrregiões com altas taxas de feminicídio correlacionadas a microrregiões vizinhas e de altas e baixas taxas de homicídio de mulheres, através do Índice Local de Moran Bivariado. Também se aplicou o modelo de varredura multivariado para múltiplos conjuntos de dados, com vistas a identificar, simultaneamente, *clusters* de feminicídio e homicídio de mulheres que coincidiram no tempo e no espaço. Foram identificados 698 feminicídios consumados no ínterim, distribuídos em 19 microrregiões de saúde. Em todo o período, as taxas de homicídio de mulheres foram maiores que as taxas de feminicídio. Verificou-se estabilidade do risco de feminicídio e tendência decrescente do risco de homicídios de mulheres entre as microrregiões de saúde de Minas Gerais. Em 11 microrregiões, o número de feminicídios superou o número de homicídios de mulheres. Os resultados evidenciaram fragilidades na definição da causa básica de óbito de pessoas do sexo feminino, afetando a qualidade do registro dos homicídios.

## Abstract (en)

This study characterizes feminicides taking place in the state of Minas Gerais, Brazil, and compare the trends in feminicide and female homicide rates across the 89 health microregions of the state. A cross-sectional ecological study was conducted using data on feminicides from 2016 to 2020, consolidated by the Minas Gerais Prosecutor’s Office. Feminicide and female homicide rates per 100,000 females were estimated according to the health microregions. Public data from the Brazilian Mortality Information System were used for the estimation of female homicide rates. An analysis was conducted on microregions with high feminicide rates correlated with neighboring microregions with high and low female homicide rates, using Bivariate Moran’s Local Indice. A multivariate scan model was also applied to multiple data sets, in order to simultaneously identify clusters of feminicide and female homicide that coincided in time and space. A total of 698 feminicides were identified during the period, distributed across 19 health microregions. During the period, female homicide rates were higher than those of feminicide. Stability in the risk of feminicide and a decreasing trend in the risk of female homicides were observed across the health microregions of Minas Gerais. In 11 microregions, the number of feminicides exceeded the number of female homicides. The results highlighted weaknesses in the determination of the underlying cause of death for females, affecting the quality of homicide records.

## Abstract (es)

El objetivo de este estudio fue caracterizar los feminicidios ocurridos en Minas Gerais, Brasil, y comparar la tendencia de las tasas de feminicidio y homicidio de mujeres en las 89 microrregiones de salud del estado. Se realizó un estudio transversal de tipo ecológico con base en datos de feminicidios consumados ocurridos entre 2016-2020, consolidados por el Ministerio Público de Minas Gerais. Las tasas de feminicidio y homicidio de mujeres se calcularon por cada 100.000 mujeres, según las microrregiones de salud. Para calcular la tasa de homicidios de mujeres, se utilizaron datos públicos del Sistema de Informaciones sobre Mortalidad. Se realizó un análisis de microrregiones con elevadas tasas de feminicidio correlacionadas con microrregiones vecinas, con altas y bajas tasas de homicidio de mujeres, utilizando el Índice Local de Moran Bivariado. También se aplicó el modelo de escaneo multivariado para múltiples conjuntos de datos, con miras a identificar, de manera simultánea, grupos de feminicidios y homicidios de mujeres que coincidieron en el tiempo y el espacio. Durante el período, se identificaron 698 feminicidios consumados, distribuidos en 19 microrregiones de salud. Durante todo el período, las tasas de homicidio de mujeres fueron más altas que las tasas de feminicidio. Se constató estabilidad en el riesgo de feminicidio y una tendencia decreciente en el riesgo de homicidios de mujeres entre las microrregiones de salud de Minas Gerais. En 11 microrregiones el número de feminicidios superó el número de homicidios de mujeres. Los resultados pusieron de manifiesto debilidades en la definición de la causa básica de muerte de las mujeres, lo que afecta la calidad del registro de homicidios.

## Introdução

A violência contra as mulheres é uma grave violação de direitos humanos e um importante problema de saúde pública ^1^. A Organização das Nações Unidas (ONU) a define como “*qualquer ato de violência de gênero que resulte ou possa resultar em dano ou sofrimento físico, sexual ou mental para a mulher, incluindo ameaças, coerção ou privação de liberdade, seja na vida pública ou privada*” ^2^ (p. 3). O resultado mais grave da continuidade das violências vivenciadas por mulheres é o óbito, especialmente por agressão interpessoal e suicídio ^3^ ^,^ ^4^.

O homicídio é compreendido como o assassinato de uma pessoa por outra com a intenção de causar morte ou grave lesão ^5^. Na Classificação Internacional de Doenças, 10ª revisão (CID-10), os códigos X85 a Y09 incluem os homicídios e as lesões interpessoais, por qualquer meio, com a intenção de ferir ou causar o óbito ^6^. Em 2022, cerca de 89 mil meninas e mulheres morreram por homicídio no mundo ^7^. Entretanto, 55% desses foram perpetrados por parceiros íntimos ou familiares ^8^, revelando que a morte violenta de mulheres deve ser compreendida como a forma mais extrema da violência baseada no gênero ^9^.

Com intuito de dar destaque às particularidades envolvendo os homicídios de mulheres, a feminista e deputada mexicana Marcela Lagarde propôs o uso do termo “feminicídio” para qualificar os homicídios de mulheres a partir de uma perspectiva de gênero ^10^. A adoção desse termo amplia o debate político ao considerar a responsabilidade do Estado no cumprimento de suas obrigações na proteção das mulheres e na garantia de seus direitos ^10^ ^,^ ^11^ ^,^ ^12^.

No Brasil, o feminicídio ganhou destaque no âmbito das políticas públicas quando, em 2015, entrou em vigor a chamada Lei do Feminicídio (*Lei nº 13.104/2015* ^13^), que o qualificou como o homicídio cometido contra a mulher por razões da condição de sexo feminino, ou seja, quando envolve violência doméstica e familiar ou menosprezo/discriminação à condição de mulher.

Apesar do avanço normativo, ainda se observam dificuldades relacionadas à quantificação, disponibilidade e análise de dados oficiais sobre essas mortes. A principal fonte de informação dos feminicídios são os registros da segurança pública, o que depende da reconstrução de um complexo quadro de circunstâncias e contextos. Essa investigação engloba a compreensão, por parte da autoridade policial, de aspectos subjetivos relacionados à vida pessoal, familiar, afetiva e profissional da vítima e do provável autor da agressão ^14^.

Ressalta-se que é a autoridade policial (ao ter conhecimento, por qualquer meio, de uma *notitia criminis* sobre uma tentativa ou morte violenta de uma mulher) quem inicia a investigação, com vistas à determinação da autoria, da materialidade e das circunstâncias do fato delituoso ^14^ ^,^ ^15^.

A atribuição para a investigação de homicídios, consumados ou tentados, é da Polícia Civil ^14^ ^,^ ^15^. Concluídas as investigações, o inquérito policial deve ser encaminhado ao Ministério Público (MP), responsável pela ação penal pública. É no ato da denúncia, realizada pelo MP, que se dá a classificação de um crime como feminicídio ou não, a partir da análise dos elementos probatórios coletados ao longo da investigação ^15^. Todo o histórico da investigação deve ser registrado, porém não há padronização para tais dados a nível nacional, o que dificulta e fragiliza a análise e a compreensão do fenômeno em todo o país ^16^.

No setor saúde, o Sistema de Informações sobre Mortalidade (SIM) fornece dados sobre a causa de óbito segundo a CID-10 ^17^. Essa classificação, apesar de não possuir código específico para o feminicídio, permite que sejam produzidas estatísticas vitais de homicídios envolvendo mulheres, porém sem levar em consideração a dimensão de gênero.

Dados do *Anuário Brasileiro de Segurança Pública* ^16^ mostram que, no Brasil, houve 929 feminicídios consumados em 2016 e, em 2021, o número registrado foi 1.341. A segurança pública de Minas Gerais também vem registrando e publicando desde 2015 os casos de feminicídio em decorrência de violência doméstica e familiar contra as mulheres. Entre 2016 e 2020, foram registrados 737 casos de feminicídio consumado no estado ^18^ ^,^ ^19^, com respectiva taxa igual a 1,4 morte por 100 mil mulheres em 2019 (mais elevada que a do Brasil - 1,2/100 mil habitantes) ^16^.

O *Atlas da Violência*, a partir dos dados do SIM, identificou 3.737 assassinatos de mulheres em 2019 no Brasil. Em Minas Gerais, a taxa de mortalidade por homicídio de mulheres foi de 2,7 óbitos/100 mil habitantes em 2019, inferior à observada no Brasil (3,5 óbitos/100 mil habitantes) ^20^.

Os dados acima demonstram que o Estado de Minas Gerais apresenta relevância quanto ao quantitativo de feminicídios contabilizados entre 2016 e 2020; ademais, a literatura sobre o tema é fragmentada e pouco aprofundada no que diz respeito às possibilidades analíticas e explicativas desse fenômeno. Nesse sentido, este estudo teve por objetivo caracterizar os feminicídios ocorridos em Minas Gerais entre 2016 e 2020, comparar as taxas de feminicídio e de homicídio de mulheres registrados nas microrregiões de saúde do estado e analisar a heterogeneidade temporal e espacial de tais crimes.

## Método

Estudo transversal do tipo ecológico, utilizando dados anuais de feminicídio consumado no Estado de Minas Gerais extraídos dos Boletins de Ocorrência (BO) registrados entre 2016 e 2020, consolidados e anonimizados no âmbito do Centro de Apoio Operacional das Promotorias de Justiça de Combate à Violência contra a Mulher (CAO-VD) do MP do Estado de Minas Gerais.

Os dados se referiram a todos os registros de homicídios com vítimas mulheres cujo BO indicava como natureza secundária a violência doméstica e familiar. O apontamento no BO sobre a violência doméstica reflete a percepção inicial das autoridades policiais a respeito da situação registrada, sendo que não há campo específico para registro de feminicídio nesse boletim. A classificação de uma situação como crime de feminicídio - ou como outro crime - se dá em momento posterior, no ato da denúncia realizada pelo MP.

Os dados de homicídio foram extraídos do SIM, sendo considerados os óbitos de mulheres cujas causas básicas compreenderam aos códigos X85 a Y09 da CID-10. Esses dados são públicos, disponibilizados no sítio eletrônico do Departamento de Informática do SUS (DATASUS; https://datasus.saude.gov.br/).

O recorte geográfico do estudo foi o Estado de Minas Gerais, que conta com 853 municípios organizados em 89 microrregiões de saúde, e que são a base territorial do planejamento da atenção à saúde ^21^.

Inicialmente, foi realizada a descrição dos feminicídios com o cálculo das frequências absolutas e relativas segundo características do feminicídio (ano do óbito, microrregiões de saúde, dia da semana da ocorrência, local da violência e meio de agressão), das mulheres (faixa etária em anos, raça/cor da pele, escolaridade, estado civil e ocupação) e, para os casos em que foi registrado apenas um autor, foram descritos o vínculo entre esse e a vítima e a situação de prisão.

Posteriormente, foram calculadas as taxas de feminicídio e de homicídio, por 100 mil mulheres, nas microrregiões de saúde de ocorrência, no período de 2016 a 2020. Para as taxas de homicídio, foram considerados os óbitos por agressão (X85 - Y09 da CID-10) de mulheres de todas as idades e a população delas para o mesmo ano. A taxa de feminicídio considerou o número de feminicídios consumados e a população de mulheres no mesmo ano. Para o cálculo das taxas, foram utilizados nos denominadores os dados de população disponibilizados *online* pelo DATASUS ^22^. Observou-se as séries temporais de ambas as taxas e se avaliou o comportamento da curva estimada de risco através de modelos aditivos generalizados (GAM, do inglês *generalized additive models*), em que as variáveis respostas (número anual de feminicídios e de homicídios de mulheres) seguiram distribuição de Poisson, com termo *offset* no preditor linear sendo o logaritmo natural da população residente feminina no mesmo ano. A interpretação foi baseada nas análises gráficas das curvas estimadas do risco de ocorrência de cada agravo e, para além da tendência temporal da série, foi avaliado o ponto de corte no tempo que indicasse uma mudança no risco estimado ^23^.

Com vistas a avaliar a distribuição geográfica das taxas supracitadas, mapearam-se as taxas observadas no período segundo microrregião de saúde. Como técnicas utilizadas para descrição e visualização de distribuições espaciais, foram utilizadas identificação de localidades atípicas (*outliers* espaciais), identificação de padrões de associação espacial (*clusters* espaciais) e indicação de diferentes regimes espaciais e outras formas de instabilidade espacial ^24^. Foi realizada análise de sub-regiões de microrregiões com altas taxas de feminicídio correlacionadas a microrregiões vizinhas, e de altas e baixas taxas de homicídios de mulheres através do Índice Local de Moran Bivariado ^18^. A matriz de vizinhança do tipo *Queen* foi utilizada, em que microrregiões são consideradas vizinhas ao compartilharem qualquer ponto de fronteira ^25^.

Para a análise da distribuição espaço-temporal dos óbitos por feminicídio e homicídio de mulheres e sua coincidência em ambos os parâmetros (espaço e tempo), aplicou-se o modelo de varredura multivariado para múltiplos conjuntos de dados, com vistas a buscar simultaneamente *clusters* de feminicídios e de homicídios de mulheres que coincidiram no tempo e no espaço no período de 2016 a 2020 e para as 89 microrregiões de saúde do Estado. As unidades espaciais de análise foram as microrregiões, categorizadas conforme o Plano Diretor de Regionalização da Secretaria de Estado de Saúde de Minas Gerais ^21^, enquanto as unidades temporais foram os anos de notificação. Utilizou-se a estatística de varredura proposta por Kulldorff ^26^, a qual permite: detectar *clusters* espaço-temporais utilizando a distribuição de probabilidade de Poisson discreta; testar significância estatística e corrigir testes múltiplos; examinar a dinâmica do evento em tempo contínuo; estimar o risco relativo para cada *cluster* considerando a população subjacente; e avaliar, simultaneamente, mais de um desfecho ^27^. Os seguintes parâmetros foram definidos para as análises: não ocorrência de sobreposição geográfica de *clusters*; e tamanho espacial máximo de cada *cluster* definido a 35% da população residente. Utilizou-se 1 a 3 anos como janela temporal e 999 simulações do método Monte Carlo com um valor de significância igual a 5% ^28^. Os *clusters* foram organizados em ordem de importância de acordo com o valor da razão de log-verossimilhança ^26^ ^,^ ^27^. A partir dos resultados, cada um foi caracterizado de acordo com as informações providas pelo software, e foram reportados apenas os significativos. Todas as análises foram realizadas nos softwares QGIS (https://qgis.org/en/site/), R (http://www.r-project.org), RStudio (https://rstudio.com/) e SaTScan (http://www.satscan.org).

O estudo utilizou dados agregados e anonimizados, sendo desenvolvido no âmbito do Projeto *Coorte Retrospectiva dos Feminicídios em Minas Gerais: Oportunidades Perdidas na Resposta à Violência contra Mulheres e Meninas no Setor Saúde*, com aprovação na Comissão Nacional de Ética em Pesquisa (CAAE nº 53271621.7.0000.5091).

## Resultados

Foram identificados 698 feminicídios consumados no Estado de Minas Gerais entre 2016 e 2020, distribuídos em 19 das 89 microrregiões de saúde. A ocorrência deles foi similar entre os dias da semana (segunda a quinta) (52%) e o final da semana (sexta, sábado e domingo) (47,9%). O principal local de ocorrência foi a residência (70,6%), seguida da via pública (24,1%). Os meios de agressão mais frequentes foram arma branca (58,5%) e arma de fogo (20,9%).

As mulheres morreram em diferentes idades, variando de 0 a 91 anos, sendo que 50% delas morreram até os 34 anos de idade (dados não demonstrados). Mais da metade (58,7%) dos óbitos envolveram mulheres de 30 a 59 anos e 31,4% ocorreram em mulheres jovens, de 15 a 29 anos; mais de 63% dessas mulheres eram da raça/cor preta ou parda e 45% era casada ou em união estável. As variáveis escolaridade e ocupação apresentaram grandes proporções de dados ignorados, de 49,1% e 67,1%, respectivamente. Cerca de 13% dessas mulheres eram do lar e 7,9% tinham ocupação classificada como serviços.

Em relação aos autores dos feminicídios, quase a totalidade dos casos (94,1%) foi cometida por um único autor. Esses possuíam idade entre 15 e 83 anos, sendo que 50% cometeram o feminicídio com 37 anos ou menos (dados não demonstrados), e 63,8% tinham entre 30 e 59 anos. Mais de 60% eram da raça/cor preta e parda, 23,3% não tinham escolaridade declarada no boletim e 46,3% eram casados/união estável. A ocupação apresentou mais de 60% de registros ignorados, e o principal vínculo do autor com a vítima foi “parceiro íntimo” (83,7%). Em 39,6% dos casos, foram localizados pela Polícia Militar de Minas Gerais (PMMG) e conduzidos até a autoridade policial, o que não ocorreu em 48,9% dos casos (Tabela 1).

**Tabela 1:** Distribuição dos casos segundo características do feminicídio, da mulher e do autor do feminicídio. Minas Gerais, Brasil, 2016 a 2020.

Características	n	%
Feminicídio		
Ano do óbito		
2016	132	18,91
2017	137	19,63
2018	129	18,48
2019	150	21,49
2020	150	21,49
Total	698	100,00
Microrregião de saúde		
Belo Horizonte/Nova Lima/Caeté	61	8,74
Ipatinga	60	8,60
Juiz de Fora	57	8,17
Contagem	52	7,45
Divinópolis	51	7,31
Montes Claros	49	7,02
Curvelo	46	6,59
Teófilo Otoni/Malacacheta	46	6,59
Governador Valadares	38	5,44
Uberaba	37	5,30
Vespasiano	31	4,44
Pouso Alegre	28	4,01
Uberlândia/Araguari	28	4,01
Poços de Caldas	25	3,58
Lavras	23	3,30
Unaí	19	2,72
Patos de Minas	18	2,58
Barbacena	17	2,44
Sete Lagoas	12	1,72
Total	698	100,00
Dia da semana		
Segunda-feira	88	12,61
Terça-feira	86	12,32
Quarta-feira	81	11,60
Quinta-feira	108	15,47
Sexta-feira	102	14,61
Sábado	115	16,48
Domingo	117	16,76
Ignorado	1	0,14
Total	698	100,00
Local da violência		
Residência	493	70,63
Via pública	168	24,07
Serviços	28	4,01
Ignorado	9	1,29
Total	698	100,00
Meio de agressão		
Arma branca	408	58,45
Arma de fogo	146	20,92
Asfixia	43	6,16
Agressão	28	4,01
Queimadura	13	1,86
Outros	5	0,72
Ignorado	55	7,88
Total	698	100,00
Mulher		
Faixa etária (anos)		
0-4	12	1,72
5-9	3	0,43
10-14	7	1,00
15-29	219	31,38
30-59	410	58,74
60 ou mais	46	6,59
Ignorado	1	0,14
Total	698	100,00
Raça/Cor da pele		
Parda	333	47,71
Branca	178	25,50
Preta	108	15,47
Ignorada	79	11,32
Total	698	100,00
Escolaridade		
Sem escolaridade	167	23,93
Ensino Fundamental completo ou incompleto	85	12,18
Ensino Médio completo ou incompleto	80	11,46
Ensino Superior completo ou incompleto	23	3,30
Ignorado	343	49,14
Total	698	100,00
Estado civil		
Casada/União estável	321	45,99
Ignorado	172	24,64
Solteira	148	21,20
Separada/Divorciada	37	5,30
Viúva	20	2,87
Total	698	100,00
Ocupação		
Ignorado	468	67,05
Do lar	90	12,89
Serviços *	55	7,88
Profissional liberal	25	3,58
Aposentada	18	2,58
Serviços domésticos	14	2,01
Trabalhadora rural	11	1,58
Desempregada	9	1,29
Estudante	8	1,15
Total	698	100,00
Autor do feminicídio		
Número de autores		
1	657	94,13
Mais de 1	41	5,87
Total	698	100,00
Faixa etária (anos)		
15-19	23	3,50
20-29	154	23,44
30-59	419	63,77
60 ou mais	35	5,33
Ignorado	26	3,96
Total	657	100,00
Raça/Cor da pele		
Parda	312	47,49
Branca	122	18,57
Ignorada	113	17,20
Preta	110	16,74
Total	657	100,00
Escolaridade		
Sem escolaridade	153	23,29
Ensino Fundamental completo ou incompleto	121	18,42
Ensino Médio completo ou incompleto	49	7,46
Ensino Superior completo ou incompleto	18	2,74
Ignorado	316	48,10
Total	657	100,00
Estado civil		
Casado/União estável	304	46,27
Ignorado	177	26,94
Solteiro	138	21,00
Separado/Divorciado	29	4,41
Viúvo	9	1,37
Total	657	100,00
Ocupação		
Ignorado	417	63,47
Serviços *	111	16,89
Trabalhador rural	48	7,31
Desempregado	29	4,41
Motorista	17	2,59
Profissional liberal	16	2,44
Aposentado	14	2,13
Estudante	5	0,76
Total	657	100,00
Vínculo com a vítima		
Parceiro íntimo	550	83,71
Familiar	42	6,39
Outro parentesco	27	4,11
Amigo/Conhecido	12	1,83
Ignorado	26	3,96
Total	657	100,00
Situação		
Sem prisão	321	48,86
Preso em flagrante	260	39,57
Suicídio	21	3,20
Ignorado	55	8,37
Total	657	100,00

Fonte: Centro de Apoio Operacional das Promotorias de Justiça de Combate à Violência contra a Mulher (CAO-VD)/Ministério Público de Minas Gerais.

* As ocupações categorizadas como Serviços incluem: ajudante, atendente, atendente de bar, autônoma, auxiliar de cozinha, auxiliar de escritório, auxiliar de produção, auxiliar de serviços gerais, balconista, cabeleireira, caixa, caixa de supermercado, comerciária, costureira, cozinheira, cuidadora, cuidadora de idosos, dançarina, gari, garota de programa, jovem aprendiz, manicure, operadora de caixa, sapateira, secretária, segurança, servente de pedreiro, serviços gerais e vendedora.

**Figura 1:**
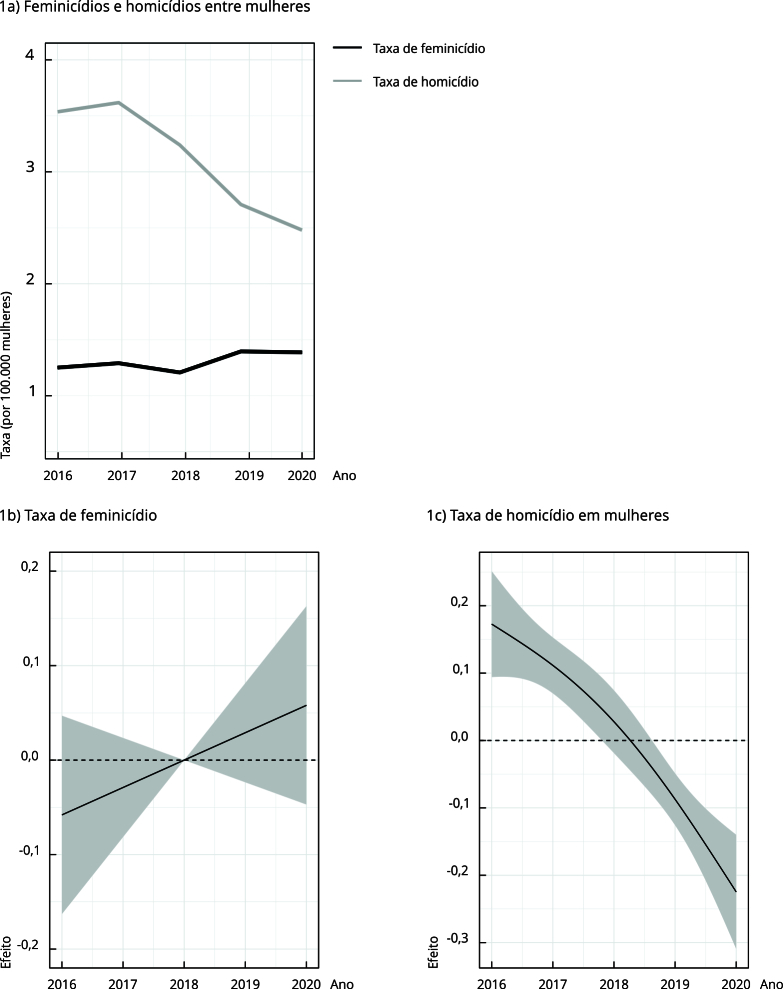
Séries temporais, curvas suavizadas dos riscos de feminicídio e homicídio de mulheres, segundo modelos aditivos generalizados e microrregiões de saúde. Minas Gerais, Brasil, 2016 a 2020.

As séries temporais das taxas de feminicídio e homicídio de mulheres mostram que, no período de estudo, as taxas de homicídio de mulheres foram maiores que as taxas de feminicídio. As taxas de homicídio de mulheres variaram entre 3,6 mortes por 100 mil mulheres no início do período e 2,4 mortes por 100 mil mulheres no seu fim, enquanto o feminicídio apresentou taxas de 0,7 morte por 100 mil mulheres em 2016 e 0,9 morte por 100 mil mulheres em 2020 (Figura 1a).

As Figuras 1b e 1c são as curvas suavizadas geradas pelos modelos GAM para o risco de ocorrência de feminicídio e homicídio de mulheres. Verificou-se estabilidade do risco de feminicídio no período, pois a curva é transpassada pelo efeito nulo em todo ele (Figura 1b), e uma tendência decrescente do risco de homicídio de mulheres no mesmo (Figura 1c) entre as microrregiões de saúde de Minas Gerais. Mesmo com séries temporais curtas e tendo sido observados intervalos de confiança amplos para a curva de risco de feminicídio, é evidente que o ponto de mudança no sentido do efeito para ambos os desfechos ocorre no ano de 2018. Dessa forma, a análise espacial de varredura prosseguiu considerando dois períodos de tempo com base nesse ponto de corte.

**Figura 2:**
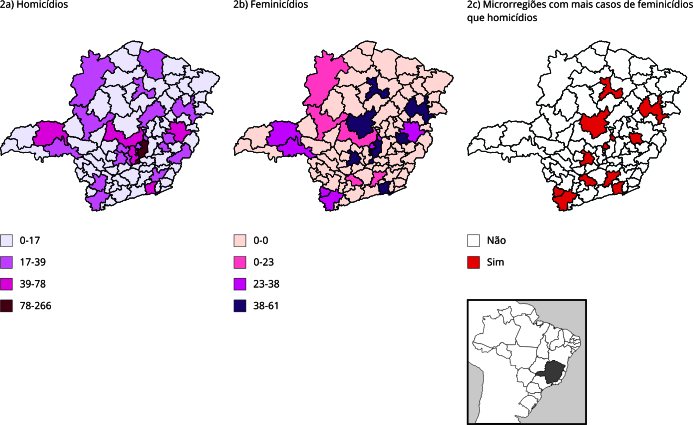
Distribuição espacial do número de casos de homicídio de mulheres, feminicídio e microrregiões de saúde com mais casos de feminicídio que homicídio de mulheres, segundo microrregiões de saúde. Minas Gerais, Brasil, 2016-2020.

A distribuição espacial do número de casos de homicídio de mulheres e feminicídio nas microrregiões de Minas Gerais no tempo avaliado mostrou que, em 11 microrregiões, o número de feminicídio superou o número de homicídio, sendo elas: Barbacena, Curvelo, Divinópolis, Ipatinga, Juiz de Fora, Lavras, Montes Claros, Poços de Caldas, Pouso Alegre, Teófilo Otoni/Malacacheta e Vespasiano (Figura 2).

Fonte: Centro de Apoio Operacional das Promotorias de Justiça de Combate à Violência contra a Mulher (CAO-VD)/Ministério Público de Minas Gerais e Sistema de Informações sobre Mortalidade (SIM) ^32^.

**Figura 3:**
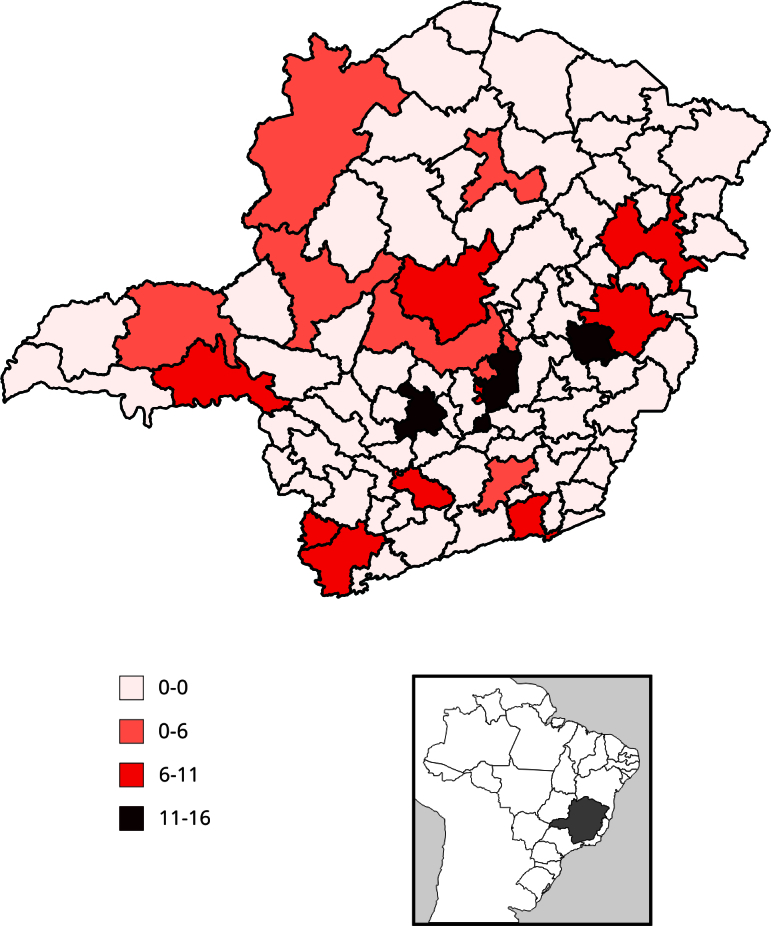
Distribuição espacial das taxas de homicídio de mulheres, segundo microrregiões de saúde. Minas Gerais, Brasil, 2016-2020.

**Figura 4:**
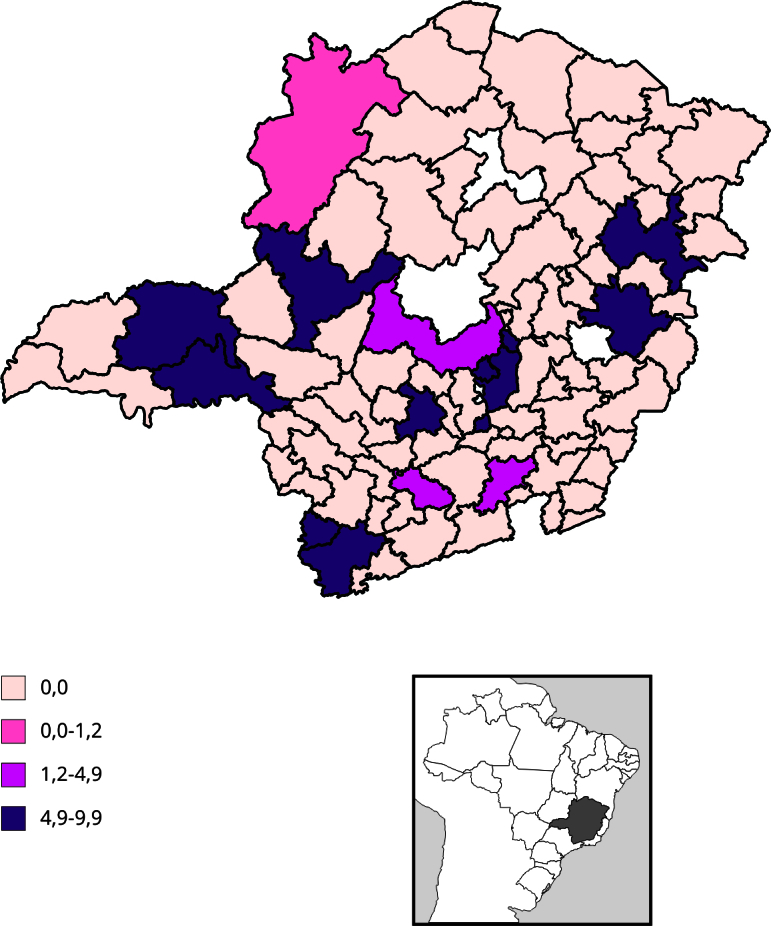
Distribuição espacial das taxas de feminicídio, segundo microrregiões de saúde. Minas Gerais, Brasil, 2016-2020.

A distribuição espacial das taxas observadas de homicídio de mulheres (Figura 3) e de feminicídio (Figura 4) mostram que Divinópolis e Belo Horizonte/Nova Lima/Caeté são microrregiões com ambas as taxas altas. Já Vespasiano, Governador Valadares, Pouso Alegre, Uberlândia/Araguari, Uberaba, Patos de Minas, Poços de Caldas, e Teófilo Otoni/Malacacheta apresentaram taxas mais altas de feminicídio.

Fonte: Sistema de Informações sobre Mortalidade (SIM) ^32^.

Fonte: Centro de Apoio Operacional das Promotorias de Justiça de Combate à Violência contra a Mulher (CAO-VD)/Ministério Público de Minas Gerais.

**Figura 5:**
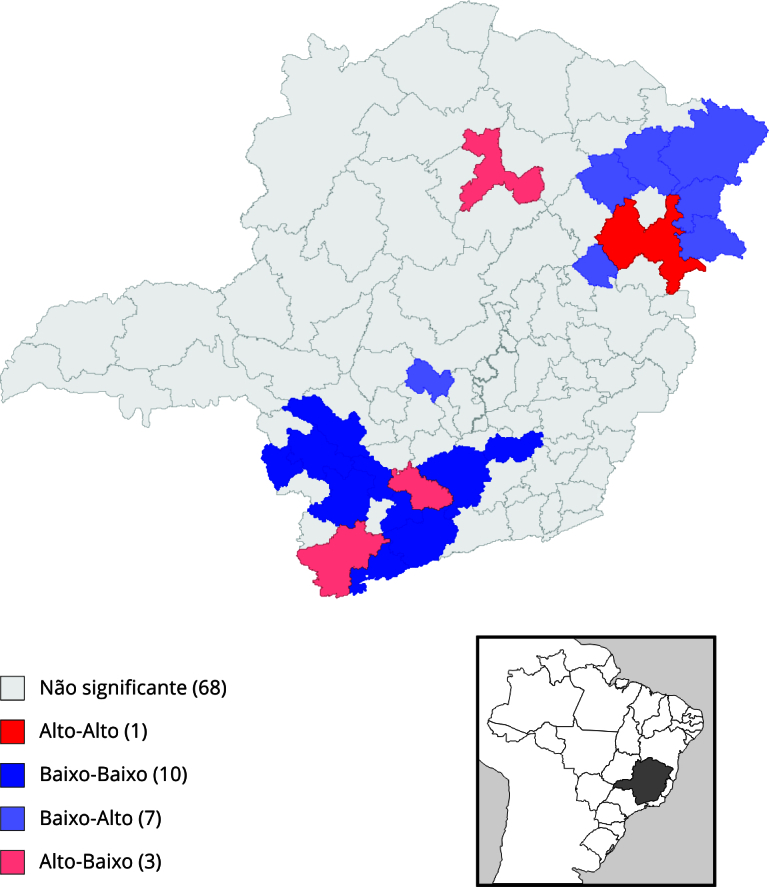
Mapeamento de quadrantes com classificação do Índice Local de Moran Bivariado e distribuição de clusters detectados via SatScan, segundo microrregiões de saúde. Minas Gerais, Brasil, 2016-2020.

O mapeamento dos quadrantes resultantes da classificação do Índice Local de Moran Bivariado se apresentou estatisticamente significativo entre as duas taxas em 21 microrregiões (Figura 5). A escala gráfica mostra os quadrantes com taxas alta-alta (áreas com altas taxas de feminicídio e homicídio de mulheres), baixa-baixa (áreas com baixas taxas de feminicídio e homicídio de mulheres), baixa-alta (áreas com baixas taxas de feminicídio, mas altas taxas de homicídio de mulheres), e alta-baixa (áreas com altas taxas de feminicídio, mas baixas taxas de homicídio de mulheres). No sul do estado, concentraram-se as microrregiões com altas taxas de feminicídio e baixas taxas de homicídio de mulheres (Pouso Alegre e Lavras) e dez microrregiões com baixa taxa de feminicídio e de homicídio de mulheres, simultaneamente (Itajubá, São Lourenço, Três Corações, São João Del Rei, Conselheiro Lafaiete, Alfenas/Machado, Três Pontas, Piumhi, Passos e São Sebastião do Paraíso). No nordeste do estado, concentraram-se as microrregiões com baixa taxa de feminicídio e alta taxa de homicídio de mulheres além de Teófilo Otoni/Malacacheta, a única microrregião que apresentou alta taxa de feminicídio e homicídio de mulheres, simultaneamente. Ao Norte, a microrregião Montes Claros apresentou alta taxa de feminicídio e baixa taxa de homicídio de mulheres comparada às regiões vizinhas (Figura 5).

Fonte: Centro de Apoio Operacional das Promotorias de Justiça de Combate à Violência contra a Mulher (CAO-VD)/Ministério Público de Minas Gerais e Sistema de Informações sobre Mortalidade (SIM) ^32^.

**Figura 6:**
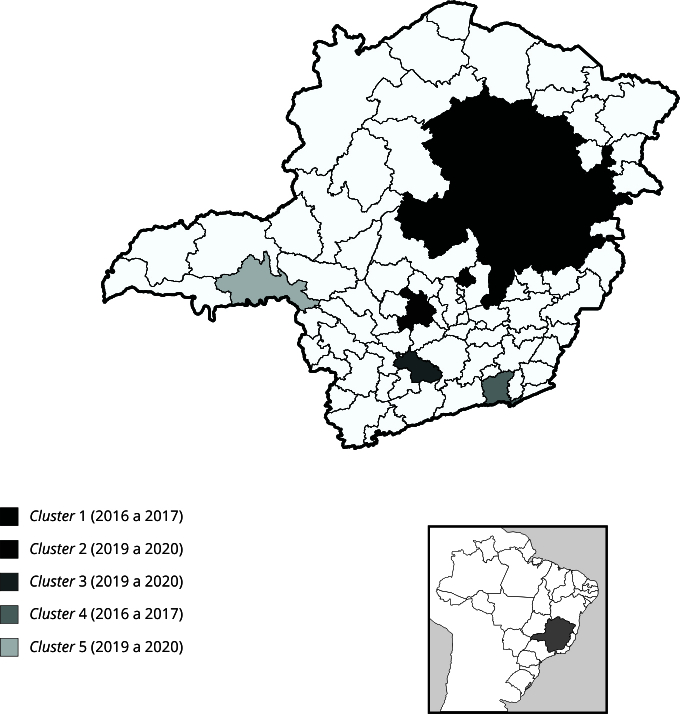
Mapeamento de *clusters* espaço-temporais simultâneos de feminicídio e homicídio de mulheres detectados via SaTScan, segundo microrregiões de saúde. Minas Gerais, Brasil, 2016 a 2020.

Cinco *clusters* espaço-temporais multivariados significativos foram detectados na análise, abrangendo dois períodos distintos: 2016 a 2017 (*clusters* 1 e 4) e 2019 a 2020 (*clusters* 2, 3 e 5). Quatro dos cinco foram simultâneos para feminicídios e homicídios de mulheres, enquanto somente um (*cluster* 3) foi caracterizado somente com feminicídios. Não foram detectados clusters significativos somente para homicídios de mulheres (Tabela 2; Figura 6).

**Tabela 2:** Resultado da análise de varredura espaço-temporal para detecção de *clusters* simultâneos de feminicídio e homicídio de mulheres. Minas Gerais, Brasil, 2016 a 2020.

*Cluster*	Localidades	Período	Casos	População	Incidência anual	RR	Razão log-verossimilhança	Valor de p
1	18	2016-2017	Feminicídio: 117 Homicídio: 120	1.599.839	Feminicídio: 3,7 Homicídio: 3,8	Feminicídio: 3,18 Homicídio: 1,23	52,531059	< 0,001
2	1	2019-2020	Feminicídio: 28 Homicídio: 12	175.818	Feminicídio: 7,8 Homicídio: 3,4	Feminicídio: 6,22 Homicídio: 1,08	27,297285	< 0,001
3	1	2019-2020	Feminicídio: 15	93.210	Feminicídio: 8,0	Feminicídio: 6,21	14,683748	< 0,001
4	1	2016-2017	Feminicídio: 28 Homicídio: 23	324.796	Feminicídio: 4,4 Homicídio: 3,6	Feminicídio: 3,43 Homicídio: 1,15	14,627580	< 0,001
5	1	2019-2020	Feminicídio: 20 Homicídio: 15	207.620	Feminicídio: 4,7 Homicídio: 3,6	Feminicídio: 3,71 Homicídio: 1,15	11,586576	< 0,05

RR: risco relativo.

Fonte: Centro de Apoio Operacional das Promotorias de Justiça de Combate à Violência contra a Mulher (CAO-VD)/Ministério Público de Minas Gerais e Sistema de Informações sobre Mortalidade (SIM) ^32^.

Nota: homicídio se refere ao óbito de mulheres cuja causa básica de óbito no SIM foram os códigos X85 a Y09 da Classificação Internacional de Doenças, 10ª revisão.

O *cluster* primário apresentou, simultaneamente, 117 casos de feminicídio e 120 casos de homicídio de mulheres em 18 microrregiões. A razão entre número de feminicídios observados e esperados foi de 2,8 vezes mais casos, enquanto a razão para homicídio de mulheres foi de 1,2. O *cluster* apresentou para o período um risco relativo de 3,18 e 1,23, respectivamente, para feminicídio e homicídio de mulheres, com taxas de 3,7 e 3,8 por 100 mil habitantes. O outro *cluster* detectado para de 2016 a 2017 (*cluster* 4) foi composto por uma microrregião (Juiz de Fora), com 28 casos de feminicídio e 23 casos de homicídio de mulheres. Foram observados 3,3 mais feminicídios do que o esperado, enquanto foram observados 1,2 mais homicídios de mulheres do que o esperado nesse *cluster*. O risco relativo foi de 3,4 e 1,1 para feminicídio e homicídio de mulheres, respectivamente (Tabela 2; Figura 6).

O período 2019 a 2020 apresentou um recrudescimento na dimensão espacial dos *clusters*. Foram detectados dois *clusters* multivariados simultâneos para feminicídio e homicídio de mulheres (*clusters* 2 e 5) e um apenas de feminicídio (*cluster* 3). Os *clusters* desse período totalizaram 63 feminicídios e 27 homicídios de mulheres, respectivamente 43,4% e 18,9% do constatado para o período 2016 a 2017 (em que se observaram 145 feminicídios e 143 homicídios de mulheres). Porém, os riscos relativos observados nos *clusters* do período 2019 a 2020 foram superiores aos do período 2016 a 2017 (Tabela 2; Figura 6).

O *cluster* 2 foi responsável por 28 casos de feminicídio e 12 casos de homicídio de mulheres em apenas uma microrregião (Divinópolis). Ocorreram seis vezes mais feminicídios observados do que o esperado, enquanto o número de homicídios de mulheres foi 1,1 vez maior que o esperado. O *cluster* apresentou risco relativo de 6,2 e 1,1, respectivamente, para feminicídio e homicídio de mulheres no período, com taxas de 7,8 e 3,4 por 100 mil habitantes. O *cluster* 5, por sua vez, apresentou 20 feminicídios (3,6 vezes mais que o esperado) e 15 homicídios de mulheres (1,1 vez mais que o esperado) em uma única microrregião (Uberaba). O risco relativo para feminicídio foi 3,7, enquanto para homicídio de mulheres foi 1,2, sendo as taxas observadas iguais a 4,7 e 3,6 casos por 100 mil habitantes, respectivamente. Por fim, o *cluster* 3 foi composto apenas pelo desfecho feminicídio. A única microrregião que o compôs (Lavras) apresentou 15 casos no período de 2019 e 2020, sendo o risco relativo igual a 6,2 e a taxa 8,0 casos por 100 mil habitantes, 6,1 vezes a mais que o esperado (Tabela 2; Figura 6).

## Discussão

O estudo analisou os 698 feminicídios registrados em Minas Gerais entre 2016 e 2020, cuja ocorrência mais frequente foi entre mulheres negras (pretas e pardas), de 30 a 59 anos, no ambiente domiciliar e com uso predominante de arma branca. O estudo inova ao apresentar as características dos autores do feminicídio, sendo a maioria parceiros íntimos, homens de 30 a 59 anos e negros (pretos e pardos).

Estudos que descreveram feminicídios na cidade de Manaus (Amazonas) e Campinas (São Paulo), também apresentaram características similares, sendo predominantes em mulheres pretas e pardas e com uso de arma branca (objeto cortante/penetrante) ^12^ ^,^ ^29^. No contexto amazônico, a maior parte dos feminicídios ocorreu em via pública ^12^, enquanto no presente estudo e em Campinas ^29^, a maioria ocorreu na residência.

Em Minas Gerais, foi registrado número duas vezes maior de óbitos por homicídio em pessoas do sexo feminino do que por feminicídios no período de 2016 a 2020. A diferença pode ser explicada pelos seguintes fatos: a qualificação do feminicídio é recente (a partir da *Lei nº 13.104/2015* ^13^), implicando em sub-registro desses casos; os homicídios de mulheres podem acontecer com motivação diversa à discriminação de gênero, especialmente em locais com alta violência comunitária; os casos de feminicídios analisados se restringiram àqueles relacionados à violência doméstica, não incluindo feminicídios cujos autores não estão no âmbito das relações íntimas, familiares ou de afeto da vítima. A escassez de dados sobre feminicídios cometidos fora do âmbito privado é uma realidade global e foi destacada em estudo publicado pelo Escritório das Nações Unidas sobre Drogas e Crime ^8^ como um dificultador para a elaboração de políticas públicas preventivas voltadas a esse tipo de crime. Um estudo realizado em Manaus mostrou que foram identificados 138 óbitos de mulheres por homicídio em 2016 e 2017, sendo que 38% foram considerados feminicídios a partir da avaliação de registros publicados na imprensa local e os demais foram relacionados ao envolvimento com o tráfico de drogas ilícitas ou com atividades criminais ^12^. Ou seja, a maior parte dos homicídios esteve relacionada à violência urbana e comunitária. No estudo realizado em Campinas, dos 26 homicídios de mulheres ocorridos em 2015, os autores caracterizaram 19 como feminicídios a partir de entrevistas com familiares da falecida ^29^. Esse padrão se repete em âmbito nacional, sendo que, em 2021, o total de óbitos de mulheres por homicídio registrados no SIM foi 3.858 ^30^, enquanto o total de feminicídios registrado pela segurança pública foi de 1.347 casos ^31^.

Em Minas Gerais, de 2016 a 2020, houve tendência decrescente na taxa de homicídio de mulheres, comportamento semelhante ao observado para o país, que registrou redução de 19,7% entre 2011 e 2021 ^30^. Contudo, considerando um período mais recente, tem-se verificado no Brasil a inversão dessa tendência, com o crescimento do homicídio de mulheres a partir de 2019 ^30^. Esses dados sugerem a necessidade da continuidade desse monitoramento no estado para acompanhar eventuais alterações desse indicador.

Com relação à taxa de feminicídio em Minas Gerais entre 2016 e 2020, verificamos estabilidade. Já os resultados da pesquisa *Feminicídio em 2023*, que utilizou dados dos BOs registrados em 2022 e 2023 pelas Polícias Civis dos estados e do Distrito Federal, demonstram aumento de 1,6% e 7% na taxa de feminicídio no Brasil e em Minas Gerais, respectivamente ^31^.

A variabilidade desses indicadores e as oscilações em suas tendências são muito mais reflexo da qualidade dos dados utilizados para elaborar as estimativas e das incorreções nas nomeações dos eventos, como no caso do feminicídio, o que impõem limites nas interpretações. Sobre isso, é importante refletir sobre a qualidade dos registros dos óbitos no SIM, sistema que fornece dados de grande confiabilidade e frequentemente utilizado em análises sobre violência. No Brasil, tem sido observado um aumento dos óbitos violentos por causa indeterminada (entre 2018 e 2019, o aumento foi de 35%) ^32^, o que pode esconder possíveis homicídios e, consequentemente, feminicídios. Essa tendência tem sido observada em diferentes estados, porém em 2019, Minas Gerais apresentou a sétima maior taxa de óbitos violentos por causa indeterminada (7,22/100 mil habitantes) ^20^. Esse fenômeno tem sido considerado um indicador da perda da qualidade dos dados do SIM ^30^, o que demonstra, por um lado, a importância de não se ignorar esses dados ao se produzirem análises sobre óbitos violentos e, por outro, a necessidade de se compreender os determinantes dessa perda de qualidade para que possam ser planejadas intervenções para o aprimoramento do processo de produção de dados do SIM.

Além disso, destaca-se que, entre 2016 e 2019, a taxa de homicídio de mulheres negras foi mais alta que a taxa entre as brancas, tanto no Brasil como em Minas Gerais ^20^. No estado, a tendência de redução dos homicídios de mulheres foi maior para as mulheres não negras (24%) em comparação às mulheres negras (20%) ^20^.

A análise espacial do número de casos de feminicídio e homicídio de mulheres segundo as microrregiões de saúde mostrou maior ocorrência de feminicídio em 11 microrregiões, sendo que em três delas (Poços de Caldas, Teófilo Otoni/Malacacheta, Vespasiano), as taxas de feminicídio também superaram as de homicídio de mulheres. Essas diferenças não esperadas podem estar relacionadas a fragilidades na definição da causa básica de óbito, afetando a qualidade do registro dos homicídios. Também é importante considerar que o número reduzido de institutos médicos legais (n = 47), concentrados em 42 microrregiões de saúde, pode afetar a qualidade dos registros das mortes violentas.

Nesse contexto, é importante ressaltar que a nomeação da morte violenta de mulheres baseada no gênero dá visibilidade ao problema, colocando-o na agenda das políticas públicas para seu enfrentamento ^11^ ^,^ ^14^. Por isso, é importante aprofundar a discussão sobre a caracterização dos óbitos motivados pela condição de gênero, qualificando as circunstâncias e problematizando o que eventualmente não vem sendo incluído nessa definição. Por exemplo, associar o feminicídio à existência de vínculo entre a vítima e o agressor limita a compreensão de que homicídios de mulheres relacionados à violência urbana e comunitária também podem ser motivados por relações desiguais de gênero. Cabe uma leitura do fenômeno do feminicídio que sempre considere o fatos dos marcadores sociais das desigualdades não operarem apenas no nível individual, requerendo, ainda, a abordagem em uma perspectiva racial, já que os resultados reiteram a maior ocorrência de casos de feminicídio entre mulheres negras (pretas e pardas).

Os registros aqui analisados apresentam limitações, sendo a série histórica pequena e dependente da identificação da violência doméstica nos registros policiais. Ainda assim, a descrição das características das mulheres e a análise espaço-temporal é inédita e pode contribuir para reflexões no campo da segurança pública e no setor saúde. Essas informações fortalecem a ideia de que o feminicídio, por ocorrer em um contexto de violência de gênero, apresenta uma dinâmica específica que o difere de outros tipos de homicídio de mulheres ^11^, reforçando a necessidade da implementação de políticas públicas específicas de enfrentamento à violência de gênero, uma vez que as causas da diminuição dos homicídios de mulheres não têm ocasionado os mesmos efeitos sobre a taxa de feminicídio, que permanece estável.

Quanto às limitações dos métodos, citam-se o curto período de tempo das séries temporais, refletido nos intervalos de confiança mais amplos para os riscos estimados em cada ponto de tempo, e a possível subestimação dos riscos encontrados para os clusters espaciais, considerando o grande número de unidades espaciais com contagens nulas. Reforça-se que essa metodologia não estima o risco para cada unidade de observação, mas para os *clusters* que foram detectados. Também, a ocorrência de grande número de zeros (ou até mesmo 1) não é um impeditivo para as análises, visto que a estatística de varredura permite considerar diferentes *clusters* (menores ou maiores) através de sua janela, que se move continuamente no espaço e/ou tempo, sem restrições impostas por delimitações geográficas administrativas e minimizando os pressupostos sobre a localização e a dimensão geográfica do aglomerado ^28^. Outros estudos também apresentam abordagens similares, utilizando conjuntos de dados com elevado números de zeros (por exemplo, casos de câncer cerebral ^33^) e o uso da metodologia em modelos multivariados (i.e., múltiplas variáveis de desfecho ^27^).

Por outro lado, percebe-se a subestimativa ocorreu em toda a região de análise, não afetando a detecção de clusters primários de forma contundente, mas apenas subestimando a magnitude dos riscos identificados nos mesmos. Pretende-se, assim, explorar outras técnicas que permitam utilizar distribuições inflacionadas de zeros em análise futuras.

Por fim, destaca-se a importância de análises que articulem dados de diferentes sistemas de informação, especificamente os registros da segurança pública e da área da saúde no caso do feminicídio. Essa análise deve ser incorporada na prática dos serviços, e não ficar restrita a estudos e equipe de profissionais da área acadêmica.
